# Comparison of Blood and Brain Mercury Levels in Infant Monkeys Exposed to Methylmercury or Vaccines Containing Thimerosal

**DOI:** 10.1289/ehp.7712

**Published:** 2005-04-21

**Authors:** Thomas M. Burbacher, Danny D. Shen, Noelle Liberato, Kimberly S. Grant, Elsa Cernichiari, Thomas Clarkson

**Affiliations:** 1 Department of Environmental and Occupational Health Sciences, School of Public Health and Community Medicine,; 2 Washington National Primate Research Center,; 3 Center on Human Development and Disability, and; 4 Departments of Pharmacy and Pharmaceutics, School of Pharmacy, University of Washington, Seattle, Washington, USA; 5 Department of Environmental Medicine, University of Rochester School of Medicine, Rochester, New York, USA

**Keywords:** brain and blood distribution, elimination half-life, ethylmercury, infant nonhuman primates, methylmercury, thimerosal

## Abstract

Thimerosal is a preservative that has been used in manufacturing vaccines since the 1930s. Reports have indicated that infants can receive ethylmercury (in the form of thimerosal) at or above the U.S. Environmental Protection Agency guidelines for methylmercury exposure, depending on the exact vaccinations, schedule, and size of the infant. In this study we compared the systemic disposition and brain distribution of total and inorganic mercury in infant monkeys after thimerosal exposure with those exposed to MeHg. Monkeys were exposed to MeHg (via oral gavage) or vaccines containing thimerosal (via intramuscular injection) at birth and 1, 2, and 3 weeks of age. Total blood Hg levels were determined 2, 4, and 7 days after each exposure. Total and inorganic brain Hg levels were assessed 2, 4, 7, or 28 days after the last exposure. The initial and terminal half-life of Hg in blood after thimerosal exposure was 2.1 and 8.6 days, respectively, which are significantly shorter than the elimination half-life of Hg after MeHg exposure at 21.5 days. Brain concentrations of total Hg were significantly lower by approximately 3-fold for the thimerosal-exposed monkeys when compared with the MeHg infants, whereas the average brain-to-blood concentration ratio was slightly higher for the thimerosal-exposed monkeys (3.5 ± 0.5 vs. 2.5 ± 0.3). A higher percentage of the total Hg in the brain was in the form of inorganic Hg for the thimerosal-exposed monkeys (34% vs. 7%). The results indicate that MeHg is not a suitable reference for risk assessment from exposure to thimerosal-derived Hg. Knowledge of the toxicokinetics and developmental toxicity of thimerosal is needed to afford a meaningful assessment of the developmental effects of thimerosal-containing vaccines.

Public perception of the safety and efficacy of childhood vaccines has a direct impact on immunization rates (Biroscak et al. 2003; Thomas et al. 2004). The current debate linking the use of thimerosal in vaccines to autism and other developmental disorders [Institute of Medicine (IOM) 2001, 2004] has led many families to question whether the potential risks associated with early childhood immunizations may outweigh the benefits (Blaxill et al. 2004; SafeMinds 2005). Thimerosal is an effective preservative that has been used in the manufacturing of vaccines since the 1930s. Thimerosal consists of 49.6% mercury by weight and breaks down in the body to ethyl-mercury and thiosalicylate (Tan and Parkin 2000). Recent reports have indicated that some infants can receive ethylmercury (in the form of thimerosal) at or above the U.S. Environmental Protection Agency (EPA) guidelines for methylmercury exposure (U.S. EPA 2005), depending on the exact vaccinations, schedule, and size of the infant (Ball et al. 2001). Clements et al. (2000) calculated that children receive 187.5 μg of ethylmercury from thimerosal-containing vaccines given over the first 14 weeks of life. According to the authors, this amount approaches or, in some cases, exceeds the U.S. EPA guidelines for MeHg exposure during pregnancy (0.1 μg/kg/day). Other estimates (Halsey 1999) have indicated that the schedule could provide repeated doses of ethylmercury from approximately 5 to 20 μg/kg over the first 6 months of life. Studies in preterm infants indicate that blood levels of Hg after just one vaccination (hepatitis B) increase by > 10-fold to levels above the U.S. EPA guidelines (Stajich et al. 2000).

The U.S. EPA guidelines for MeHg (U.S. EPA 2005) are based on several decades of studies of humans and animal models of developmental toxicity (Burbacher et al. 1990a; National Research Council 2000). Because few data exist for ethylmercury, the use of the MeHg guidelines would seem appropriate if the two compounds have similar toxicokinetic profiles and neurodevelopmental effects. The results from the few studies that have provided a direct comparison of these two compounds have been reviewed recently by Magos (2003), who concluded that *a*) Hg clears from the body faster after the administration of ethylmercury than after the administration of MeHg; *b*) the brain-to-blood Hg concentration ratio established for MeHg will overestimate Hg in the brain after exposure to ethylmercury; and *c*) because ethylmercury decomposes faster than MeHg, the risk of brain damage is less for ethylmercury than for MeHg. These conclusions are based on only a few studies, none of which included measurements of both blood and brain Hg levels in infant subjects.

We initiated the present study in order to directly compare the blood and brain levels of Hg in infant nonhuman primates exposed orally to MeHg or via intramuscular (im) injections of vaccines containing thimerosal. Nonhuman primates have been used extensively in previous studies of MeHg toxicokinetics and developmental neurotoxicity (Burbacher et al. 1986, 1990b; Gunderson et al. 1986, 1988; Rice and Gilbert 1982, 1990, 1995; Stinson et al. 1989; Vahter et al. 1994, 1995). The routes of administration (oral for MeHg and im injection for thimerosal-containing vaccines) were chosen to mimic the two routes of Hg exposure for humans. The dosages and schedule of administration of Hg were chosen to be comparable with the current immunization schedule for human newborns, taking into consideration the faster growth (~ 4 to 1) of the macaque infant (Gunderson and Sackett 1984). The results of the present study provide important new information regarding the comparative toxicokinetics of these two compounds in newborns and infants.

## Materials and Methods

### Subjects.

Forty-one infant *Macaca fascicularis* born at the Washington National Primate Research Center’s Infant Primate Research Laboratory were used in the study. The birth weights of the infant monkeys were within the normal range for this species; the average birth weight was 341 g (range, 255–420 g). Infants were weighed daily throughout the study, and any clinical problems were recorded.

### Mercury dosing schedule.

The Hg dosing schedule is shown in Table 1. Infants were assigned to one of three exposure groups at birth. Seventeen infant monkeys assigned to the thimerosal group were given the typical schedule of vaccines for human infants (Table 1). Thimerosal (Omicron Quimica S.A., Barcelona, Spain), dissolved in saline, was mixed with thimerosal-free vaccines to yield a final concentration of 4, 8, or 20 μg/mL Hg, depending on the vaccine and the age of the infant. The total dose of Hg administered via the vaccines was 20 μg/kg on day 0 and at 7, 14, and 21 days of age. A dose of 20 μg/kg was chosen based on the range of estimated doses received by human infants receiving vaccines during the first 6 months of life.

Seventeen infant monkeys assigned to the MeHg group were given MeHg hydroxide (MeHgOH, 97% pure; Alfa Aesar, Johnson Matthey Co., Ward Hill, MA) dissolved in water to a concentration of 20 μg Hg/mL. MeHg was administered to infant monkeys via oral gavage at a dose of 20 μg/kg on their day of birth (day 0) and at 7, 14, and 21 days of age.

Seven infant monkeys were assigned to a control group. These monkeys did not receive any gavages or im injections. Infants were assigned to the three groups on a semirandom basis, in order to balance sex ratios and average birth weights across groups.

### Blood draw schedule.

Blood was drawn from the saphenous vein of all infant monkeys at birth (before any Hg exposure). Blood was also drawn 2, 4, and 7 days after the initial Hg exposure (day 0) and after subsequent exposures on days 7 and 14. Depending on the sacrifice group, blood was drawn up to 28 days after the final exposure on day 21 to further characterize the washout kinetics of Hg (Table 1).

### Sacrifice schedule.

Infants were sacrificed 2, 4, 7, or 28 days after their last Hg exposure on day 21 (Table 1). Infants were sedated with an im injection of ketamine (10 mg/kg) and atropine (0.4 mg/kg) and then given an intravenous overdose of Nembutal (20 mg/kg; Abbott Labs, North Chicago, IL). Autopsy personnel from the primate center drew blood and removed the brain and other organs for analysis. The autopsy typically lasted approximately 1 hr.

The numbers of monkeys at each sacrifice day for both the MeHg and thimerosal groups were as follows: day 23 (2 days after most recent dose), *n* = 4; day 25 (4 days after most recent dose), *n* = 4; day 28 (7 days after most recent dose), *n* = 5; and day 49 (28 days after most recent dose), *n* = 4. The seven control monkeys were assigned sacrifice days as follows: day 23, *n* = 3; day 25, *n* = 1; day 28, *n* = 2; and day 49, *n* = 1 (Table 1). Monkeys were assigned to sacrifice groups at birth on a semirandom basis that balanced sex ratios and average birth weights across groups.

### Blood and brain Hg measurements.

Blood samples were prepared for Hg analysis by diluting them with an equal volume of 1% wt/vol NaCl solution. Aliquots were removed for Hg determination without digestion. One drop of antifoam reagent was added to the aliquot at the time of the analysis.

Half brain samples were fixed in formaldehyde before analysis. Samples of the fixative were analyzed to check for Hg content. The tissue was removed from the jar and blotted dry. A homogenate of the brain in 1% NaCl was prepared using a Polytron homogenyzer PT 10-35 (Brinkmann Instruments, Westbury, NY) while keeping the sample in an ice slurry. An aliquot of the homogenate was digested with 1 mL 1% wt/vol cysteine and 2 mL 45% NaOH by heating at 95°C for 10–15 min. Digest was allowed to cool and then diluted to volume by addition of 7 mL 1% wt/vol NaCl. The digests were kept in an ice slurry until analysis. Aliquots were removed for Hg determination. One drop of antifoam was added to the aliquot at the time of the analysis.

Total Hg concentrations in blood and total and inorganic Hg concentrations in brain were measured using a procedure adapted from Greenwood et al. (1977). The method determines total Hg and its inorganic fraction (Magos and Clarkson 1972). Cadmium chloride in the presence of stannous chloride at high pH breaks the Hg–carbon bond with the subsequent reduction of Hg^2+^ to Hg^0^; the latter is then measured by cold vapor atomic absorption at 254 nm with a Hg monitor (Laboratory Data Control, model 1235; Thermo Separation Products, Waltham, MA). Inorganic Hg is determined by the addition of SnCl_2_ in the absence of cadmium chloride. Concentration of organic Hg was calculated from the difference between the measured total and inorganic Hg concentrations. The original concentration of SnCl_2_ used for the Magos method (Magos and Clarkson 1972) was modified to prevent the decomposition of the ethylmercury during assay (Magos et al. 1985). To measure Hg in aqueous solution of thimerosal, the amount of SnCl_2_ was reduced from 100 μg to 50 μg/aliquot analyzed. For tissue homogenate samples, 500 μg SnCl_2_ was added to each aliquot. All reagents used for preparation and analysis of the samples were of analytical grade.

Quality control was assured by analysis of reference samples before each assay run. Fisher Mercury Reference Standard Solution (SM114-100, certified 1,000 ppm ± 1%; Fisher Scientific, Hampton, NH) was used as a stock solution. Working standards of 30 and 10 ng Hg/mL were made daily from appropriate dilutions of the stock solution. In addition, the following certified reference materials were analyzed daily before analysis of the samples: trace elements in whole blood (Seronorm Trace Elements, Certified Reference Material 201605, 6.8–8.5 μg/L; Accurate Chemical & Scientific Corp., Westburg, NY), and trace elements in human hair (Certified Reference Material 397, 12 μg/g ± 0.5; Commission of the European Communities, Geel, Belgium). The detection limit of the instrument was estimated to be 0.75 ng Hg per aliquot used for analysis.

### Data analysis.

The mean total blood Hg concentration data from both the oral MeHg and im thimerosal groups (*n* = 17 in each) were analyzed using the compartmental module of the pharmacokinetic modeling software SAAM II (SAAM Institute, Seattle, WA).

The accumulation and washout of total blood Hg concentration–time data from the MeHg monkeys were well described by a one-compartment model featuring a first-order absorption process. Regression fit of the data to the model yielded estimates of the absorption rate constant (*k*_*a*_), elimination rate constant (*K*), and an apparent volume of distribution (*V*/*F*; *F* is the implicit bioavailability term). Half-lives (*T*_1/2_) corresponding to each of the rate constants were calculated by dividing ln 2 by the rate constant estimate. Blood clearance (Cl/*F* ) was derived from the product of *K* and *V*/*F*.

A one-compartment model failed to provide a satisfactory fit of the mean total blood Hg concentration–time data from the thimerosal monkeys. The model overpredicted the blood concentration during accumulation; at the same time, it underpredicted the blood concentration during washout rate (i.e., overpredicted washout rate). Further examination of a scatter plot of the individual monkey data suggested a biphasic pattern in the washout of Hg from the blood after the last dose. Accordingly, we attempted a regression fit of the mean total blood Hg concentration data with a two-compartment model. This yielded a much better visual fit of the data, with minimal change in the objective function and Akaike information criterion. The two-compartment parameter estimates from the regression analysis included the absorption rate constant (*k*_*a*_), rate constants for Hg transfer from the central to the peripheral compartment (*k*_12_) and the return from the peripheral to the central compartment (*k*_21_), the elimination rate constant from the central compartment (*k*_10_), and the apparent volume of the central compartment (*V*_*c*_/*F* ). From these primary parameters, we further estimated the apparent distribution volume at steady state (*V*_*ss*_/*F* ) and the peripheral volume referenced to blood concentration (i.e., *V*_*p*_ = *V*_*ss*_ – *V*_*c*_). The initial and terminal rate constants and half-lives (*T*_1/2,α_and *T*_1/2,β_) for the biexponential decline of total blood Hg concentration were estimated by standard formulas (Gibaldi and Perrier 1982). Blood clearance was computed by the product of *V*_*c*_ and *k*_10_. For both the MeHg and thimerosal model fits, a fractional SD of 0.1 was used as the weighting scheme.

The washout half-life of total and organic Hg in the brain of both the oral MeHg and im thimerosal groups was estimated by regression fit to a monoexponential model using WinNonlin software (Pharsight Corp., Mountain View, CA). One of the day 28 brain samples from the MeHg exposure group had a spuriously high total Hg concentration, that is, a concentration of 151 ng/g, which is more than 50% higher than the other samples obtained on day 28 (71–90 ng/mL) and higher than those observed at the earliest sacrifice time at day 2 (75–129 ng/g). The unreasonably high concentration is most likely due to contamination of the sample. Therefore, data from this brain and its corresponding blood were excluded from the regression analysis. The average brain-to-blood concentration ratio was also calculated using data from the earliest sacrifice duration (2 days). Because of different washout half-lives in blood and the brain, brain-to-blood concentration ratio is expected to vary with the duration of washout. Samples at day 2 offered the best measure of the extent of uptake of Hg species into the brain that are least confounded by differences in their clearance rate.

Between-group statistical comparisons of the rate of washout of total Hg in blood, as well as total and organic concentrations in the brain, were accomplished through multiple regression analysis as implemented in the PROC GLM subroutine in SAS (version 9.1; SAS Institute, Cary, NC). PROC GLM performs multiple regression within the framework of general linear models and can accommodate missing data or sparse sampling and confounding from correlations between repeated measures. Hence, it is able to provide tests of hypotheses for the effects of time and group using blood and brain data obtained from sacrifice of individual animals at varying times during washout. Log-transformed blood or brain Hg concentrations in animals from both the MeHg and thimerosal groups were entered as the dependent variable. The independent variables consisted of sampling time, group (MeHg = 0, thimerosal = 1), and a time-by-group interaction. Once the overall significance of the regression model was verified, the significant sources of variation (i.e., time, group, and time by group) were identified. A difference in the rate of washout of Hg in blood or brain between groups was indicated by a significant regression coefficient for time-by-group interaction. If there was no evidence for interaction, a significant decline in blood or brain Hg concentration over time for each group was assessed by the *t*-statistic associated with the estimated regression coefficient for time.

The following statistical comparisons of the washout rate of Hg were also undertaken: total Hg in blood versus total Hg in the brain, total Hg in blood versus organic Hg in the brain, and total Hg versus organic Hg concentration in the brain. The difference between the pair of log-transformed Hg concentrations for each animal sacrificed at the various times was calculated. Individual difference values in both groups were then entered as the dependent variable in the regression model. The independent variables were time, group, and time-by-group interaction. A significant regression coefficient for the time variable indicates that the paired-log concentration difference (or the concentration ratio) varied with time; that is, the two concentration measures (e.g., blood and brain) did not decline in parallel with time.

## Results

### Growth and health status.

**Figure 1:**
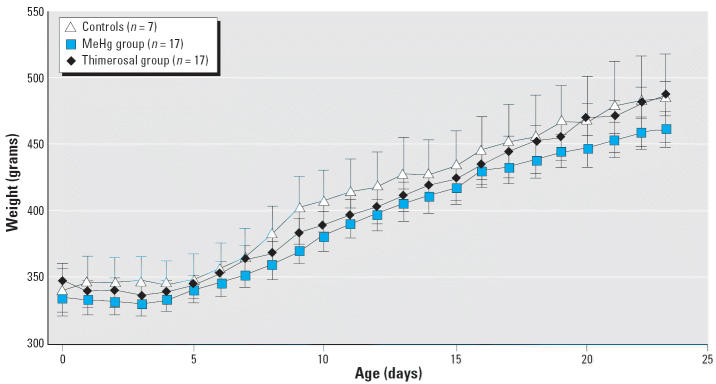
Weight gain of infant monkeys during study. Error bars indicate SE.

The weights of infant monkeys during the study are shown in Figure 1. We found no significant differences in the weight gain across the three groups (*p* > 0.10, all comparisons); the average weight gain during the first 23 days of life was 135 g. The brain weights at sacrifice and brain-to-body weight ratios are shown in Table 2; we found no significant differences in brain weights or brain-to-body weight ratios across the three groups (*p* > 0.10, all comparisons). Also, no serious medical complications were observed in any of the monkeys.

### Oral MeHg kinetics.

The total blood Hg concentrations at 2 days (observed peak) after the first dose ranged from 8 to 18 ng/mL across the monkeys, that is, a 2-fold variation. Progressive accumulation of total blood Hg was observed over the three subsequent doses of MeHg, such that the peak total blood Hg concentrations after the fourth dose were about 3-fold higher (30–46 ng/mL). The interanimal variation in blood Hg concentrations remained at about 2-fold during accumulation. Blood Hg persisted through the entire period of washout and was readily measurable in all four monkeys in the 28 day sacrifice group (16–21 ng/mL). This is consistent with previous reports of an elimination *T*_1/2_ > 20 days for MeHg in adult *M. fascicularis* (Stinson et al. 1989; Vahter et al. 1994, 1995) and explains the minimal decline (< 20%) in blood Hg concentrations during the weekly intervals between MeHg doses.

**Figure 2:**
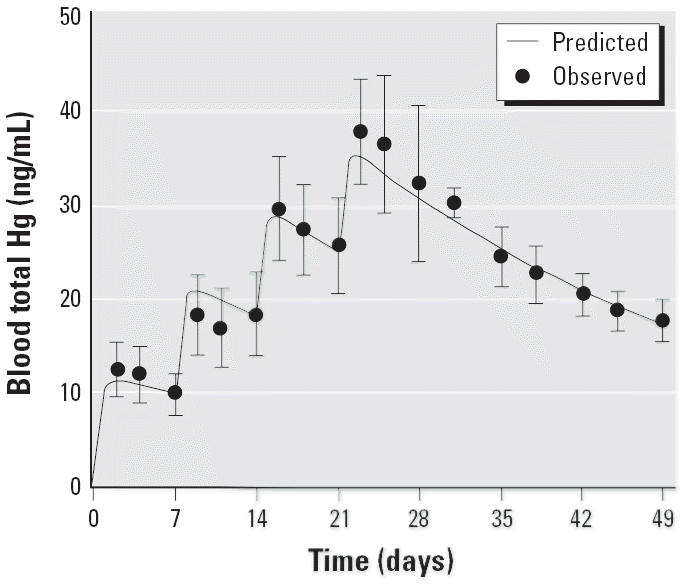
Comparison of predicted and observed mean blood total Hg concentrations during and after four weekly oral doses (20 μg/kg) of MeHg. Error bars indicate SD.

The time course of total blood Hg was fitted to a one-compartment model. Figure 2 shows the excellent regression fit of the mean blood concentration–time data. Table 3 presents parameter estimates from the one-compartment model fit of the mean blood Hg concentration–time data. The distribution volume of total Hg after MeHg administration is estimated to be 1.7 L/kg, or about 20 times the blood volume (~ 8%). This means that only 1/20th of the body burden of Hg is confined to the vascular space. This is consistent with the known extensive extravascular distribution of Hg after MeHg exposure in primates and agrees with previous estimates of Hg distribution volume in adult *M. fascicularis* (Stinson et al. 1989). The elimination *T*_1/2_ of total blood Hg is 21.5 days, which agrees with reported estimates in adult *M. fascicularis* (Stinson et al. 1989; Vahter et al. 1994, 1995). The blood clearance is estimated at 46.1 mL/day/kg, well within the range of clearance values observed earlier in adult *M. fascicularis* (Stinson et al. 1989). It appears that the systemic disposition kinetics of MeHg are the same between infant and adult *M. fascicularis*, that is, no change during development.

**Figure 3:**
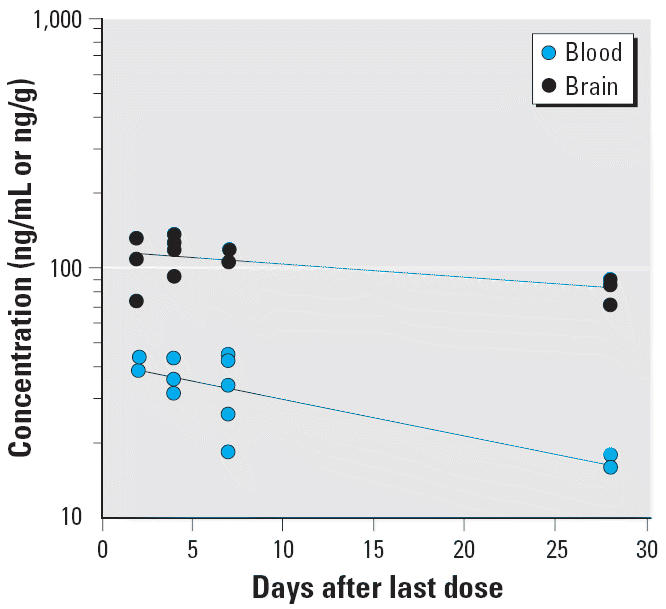
A semilogarithmic plot of washout of total Hg in blood and the brain after four weekly oral doses (20 μg/kg) of MeHg. The data were collected from groups of infant monkeys sacrificed 2, 4, 7, and 28 days after the last dose. The lines represent nonlinear regression fit of the data to a monoexponential model; the regression estimate (± SE) of *T*_1/2_ is *T*_1/2_ = 19.1 ± 5.1 days (*r* = 0.81) for blood and *T*_1/2_ = 59.5 ± 24.1 days (*r* = 0.59) for brain.

A plot of the blood and brain total Hg concentration data from the monkeys sacrificed at various times during the washout period is shown in Figure 3. There was a significant decrease in total Hg from the blood during the washout period (*p* < 0.01). The apparent *T*_1/2_ for total Hg in blood is 19.1 ± 5.1 days (± SE of regression estimate). The decrease in total Hg in the brain over time was marginally significant (*p* < 0.07), with an apparent *T*_1/2_ of 59.5 ± 24.1 days. The *T*_1/2_ for total Hg in brain was significantly longer than the *T*_1/2_ for total Hg in blood (*p* = 0.05) for the MeHg-exposed monkeys. The *T*_1/2_ for total Hg in brain is also longer than the previously reported washout *T*_1/2_ from the brain for adult *M. fascicularis* (37 days; Vahter et al. 1994, 1995). It should be noted that the relatively high SE of the half-life estimates for the brain reflects the large interanimal variation in Hg concentrations at each sampling time, limited number of data points, and the short duration of sacrifice relative to the washout half-life. The concentration of total Hg in the brain is 1.7- to 3-fold higher than in the blood (mean ± SE of 2.5 ± 0.3) 2 days after the last MeHg dose. This brain-to-blood concentration ratio increased as the duration between the last dose and the sacrifice lengthened. The ratio ranged from 3.9 to 7.4 at 28 days after the last exposure. The time dependence for the brain-to-blood ratio (*p* = 0.06) is primarily due to the difference in the washout *T*_1/2_ between total Hg in the blood and brain. The average brain-to-blood ratio for these infant monkeys at day 2 after the last MeHg dose (2.5 ± 0.3) is slightly lower than previously reported values (3–5) for adult macaque and squirrel monkeys over various durations of washout (Berlin et al. 1975; Stinson et al. 1989; Vahter et al. 1994). Although the cited differences in brain uptake and clearance of MeHg between adult and infant monkeys may be attributed to the effects of postnatal brain growth and development, it may also be related to variation in exposure regimen between studies.

**Figure 4:**
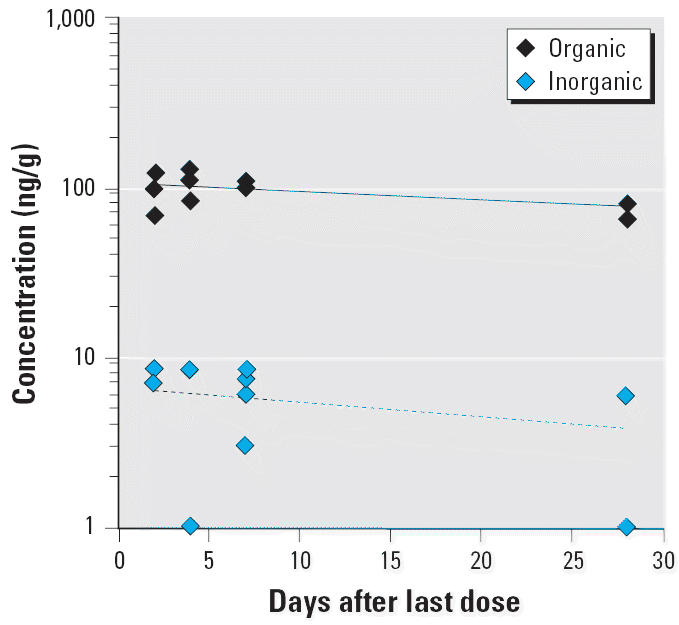
A semilogarithmic plot of the washout of organic and inorganic Hg in the brain after four weekly oral doses (20 μg/kg) of MeHg. The data were collected from groups of infant monkeys sacrificed at 2, 4, 7, and 28 days after the last dose. The lines represent nonlinear regression fit of the data to a monoexponential model. The regression estimate (± SE) for organic Hg is *T*_1/2_ = 58.4 ± 25.0 days (*r* = 0.57). The half-life of inorganic Hg is too long (> 120 days) to be accurately estimated from the present data (i.e., *r* is not significantly different from 0).

A plot of the organic and inorganic Hg concentrations in the brain of MeHg-exposed monkeys sacrificed at various times during the washout period is shown in Figure 4. The decrease in organic Hg in the brain over time was not statistically significant (*p* = 0.17). The apparent *T*_1/2_ for the washout of organic Hg from the brain was 58.4 ± 25.0 days, close to the *T*_1/2_ for total Hg. The concentration of inorganic Hg in the brain samples was below the quantifiable limit of the assay (7 ng/mL) in 8 of 17 MeHg-exposed monkeys. The average concentration of inorganic Hg for those monkeys with values above the detection limit (*n* = 10) did not change significantly over 28 days of washout and was approximately 7–8 ng/mL (Figure 4). Inorganic Hg represented only 6–10% of total Hg in the brain. These values are consistent with previously reported data in adult *M. fascicularis* (Vahter et al. 1994, 1995).

### Intramuscular thimerosal kinetics.

The initial total Hg concentrations in the day 2 blood samples, which ranged from 6 to 14 ng/mL, are comparable with the concentrations observed in the oral MeHg group. These blood levels are also similar to those reported in preterm human infants receiving 12.5 μg Hg from a hepatitis B vaccine (Stajich et al. 2000). Blood Hg concentrations declined relatively rapidly (by > 50%) between doses. As a result, there was minimal accumulation in blood Hg concentrations during weekly dosing. Also, blood Hg concentrations dropped below the detection limit of the assay in some animals by day 10 after the last vaccine injection.

**Figure 5:**
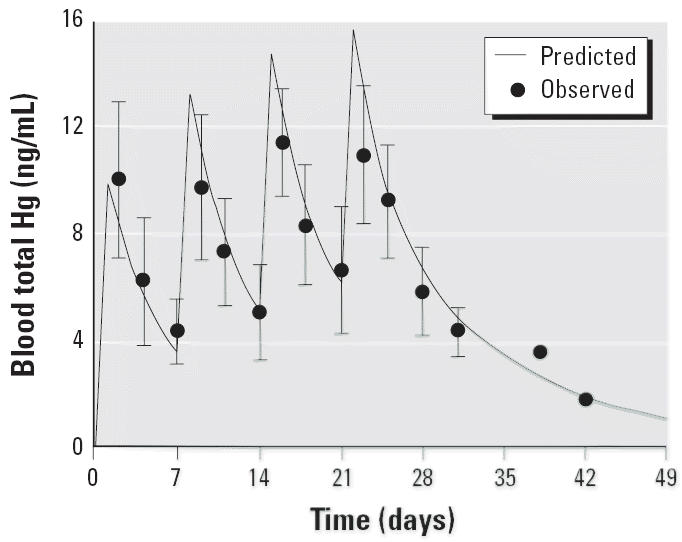
Comparison of predicted and observed mean blood total Hg concentration during and after four weekly im injections of vaccine containing thimerosal (20 μg/kg Hg). Error bars indicate SD.

The time course of total blood Hg concentrations was best described by a two-compartment model; that is, the disposition kinetics are biphasic, with a rapid initial phase followed by a slower terminal phase of clearance. Table 4 presents the parameter estimates derived from the two-compartment model analysis. A comparison of the model prediction and the observed blood concentration data is shown in Figure 5. The model predicted some accumulation in peak blood Hg concentrations and minimal accumulation in trough concentrations. Because blood concentration data were not available before day 2, the predicted peak concentrations are extrapolations and should be viewed with caution. The initial volume of distribution in the central compartment was 1.7 L/kg, which is comparable with the overall distribution volume for oral MeHg. The initial and terminal blood half-lives were 2.1 and 8.6 days, respectively. Mercury derived from thimerosal is eliminated much more rapidly than MeHg. The steady-state volume of distribution (i.e., *V*_*ss*_ or the fully equilibrated volume) was estimated to be 2.5 L/kg, which is 50% larger than the initial distribution volume (i.e., *V*_*c*_). Hence, the effective peripheral compartment volume at steady state is about 0.8 L/kg. Alternately, this means that, at steady state, partitioning of the body burden of Hg between the tissue regions associated with the central and peripheral compartments is about 2:1. The blood clearance of total Hg was estimated to be 248 mL/day/kg, which is 5.4-fold higher than the estimate for oral MeHg.

**Figure 6:**
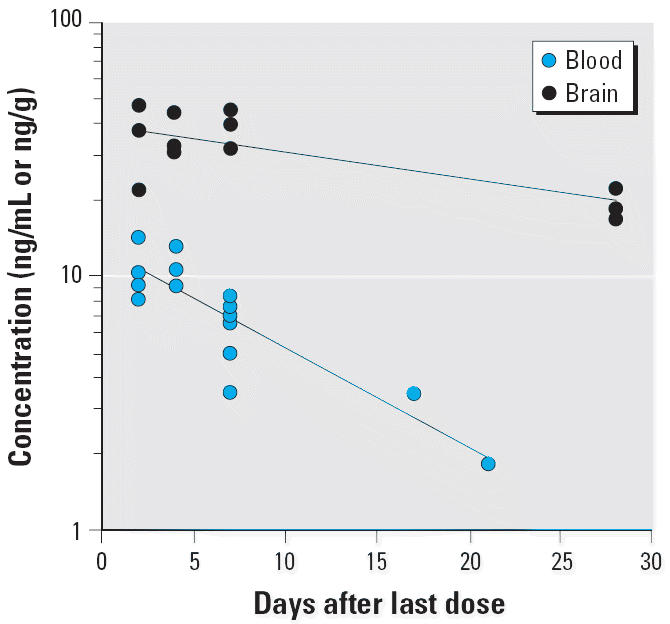
A semilogarithmic plot of washout of total Hg in blood and the brain after four weekly im injections of vaccine thimerosal (20 μg/kg Hg). The data were collected from groups of infant monkeys sacrificed at 2, 4, 7, 10, 17, and 21 days after the last dose. The lines represent nonlinear regression fit of the data to a monoexponential model. The regression estimate (± SE) of *T*_1/2_ is 24.2 ± 7.4 days (*r* = 0.74) for brain and 6.9 ± 1.7 days (*r* = 0.82) for blood.

Figure 6 presents a scatter plot of the blood and brain total Hg concentration data for monkeys sacrificed at various times during the washout. There was a significant decrease in total Hg concentration in the blood during the washout period (*p* < 0.01). The apparent *T*_1/2_ for total Hg in blood is 6.9 ± 1.7 days. There was also a significant decrease in total Hg concentration in the brain over time (*p* < 0.01), with an apparent *T*_1/2_ of 24.2 ± 7.4 days. The *T*_1/2_ for total Hg in brain was significantly longer than the *T*_1/2_ for total Hg in blood (*p* < 0.01) for the thimerosal-exposed monkeys. In addition, the *T*_1/2_ for total Hg in blood and brain for these monkeys (6.9 ± 1.7 days and 24.2 ± 7.4 days, respectively) are significantly shorter (*p* < 0.01) than the *T*_1/2_ for total Hg in blood and brain for the MeHg monkeys (19.1 ± 5.1 days and 59.5 ± 24.1 days). The concentration of total Hg in the brain of the thimerosal-exposed monkeys is 2.6- to 4.6-fold higher than in the blood (mean ± SE, 3.5 ± 0.5) at 2 days after the last injection. Again, this ratio increased as the sacrifice was performed at longer durations from the last dose, primarily due to the difference in the half-lives of total Hg in the blood and brain.

**Figure 7:**
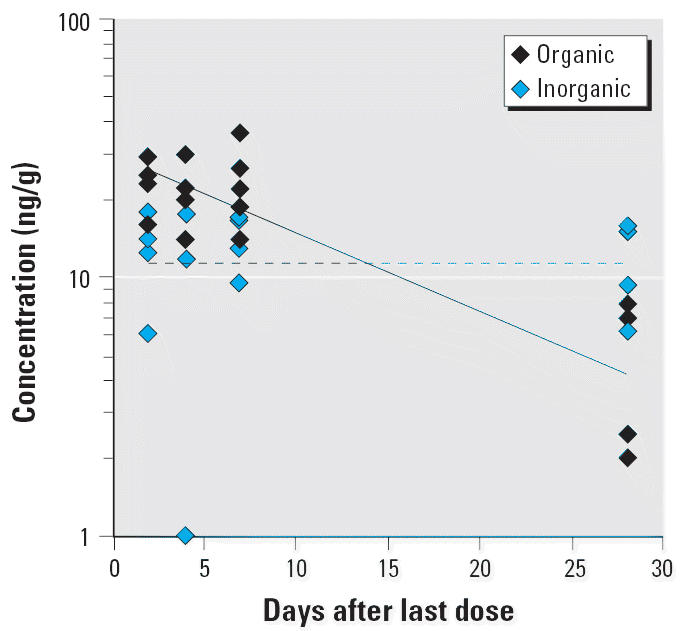
A semilogarithmic plot of washout of organic and inorganic Hg in the brain after four weekly im injection of vaccines containing thimerosal (20 μg/kg Hg). The data were collected from groups of infant monkeys sacrificed at 2, 4, 7, and 28 days after the last dose. The lines represent nonlinear regression fit of the data to a monoexponential model. The regression estimate (± SE) of *T*_1/2_ for organic Hg is *T*_1/2_ = 14.2 ± 5.2 days (*r* = 0.76). The half-life of inorganic Hg is too long (> 120 days) to be accurately estimated from the present data (i.e., *r* is not significantly different from 0).

A plot of the organic and inorganic Hg concentrations in the brain of thimerosal-exposed infant monkeys sacrificed at various times during the washout period is shown in Figure 7. There was a significant decrease in organic Hg in the brain over the washout period (*p* < 0.01). The apparent *T*_1/2_ for the washout of organic Hg from the brain was 14.2 ± 5.2 days, which is significantly shorter than the *T*_1/2_ for total Hg in brain (*p* < 0.01). The inorganic form of Hg was readily measurable in the brain of the thimerosal-exposed monkeys. The average concentration of inorganic Hg did not change across the 28 days of washout and was approximately 16 ng/mL (Figure 7). This level of inorganic Hg represented 21–86% of the total Hg in the brain (mean ± SE, 70 ± 4%), depending on the sacrifice time. These values are considerably higher than the inorganic fraction observed in the brain of MeHg monkeys (6–10%).

## Discussion

There are notable similarities and differences in the kinetics of Hg after oral administration of MeHg and im injection of thimerosal in vaccines. The absorption rate and initial distribution volume of total Hg appear to be similar between im thimerosal and oral MeHg. This means approximately equal peak total blood Hg levels after a single exposure to either MeHg or thimerosal or after episodic exposures that are apart by longer than four elimination half-lives (i.e., > 80 days for MeHg or > 28 days for thimerosal). Studies in preterm and term human infants have reported similar results (Stajich et al. 2000). Infants receiving 12.5 μg Hg from a single hepatitis B vaccine had blood Hg levels at 48–72 hr, consistent with what would be anticipated after an equivalent dose of MeHg.

Although the initial distribution volume of total Hg is similar for the two groups, a biphasic exponential decline in total blood Hg is observed only after im injections of thimerosal. This suggests continual distribution into and localization in tissue sites over time. It is relevant to note that the kidney-to-blood concentration gradient of total Hg is much higher in the thimerosal monkeys than in the MeHg monkeys (mean ± SE, 95.1 ± 10 vs. 5.8 ± 0.6). The second slower phase of washout could also represent the gradual biotransformation of ethylmercury (the presumed principal organic form of Hg after thimerosal administration) to Hg-containing metabolites that have a different tissue distribution or are more slowly eliminated. Further investigations of the disposition fate of thimerosal-derived Hg should address these issues.

Total Hg derived from im thimerosal is cleared from the infant *M. fascicularis* much more quickly than MeHg. The washout *T*_1/2_ of total blood Hg after im injections of thimerosal in vaccines is much shorter than the *T*_1/2_ of MeHg (6.9 vs. 19.1 days). These results support the earlier conclusion of Magos (2003) that Hg is cleared from the body faster after the administration of ethylmercury than after the administration of MeHg. More interestingly, the washout blood Hg *T*_1/2_ in the thimerosal-exposed infant macaques (7 days) is remarkably similar to the blood Hg *T*_1/2_ reported for human infants injected with thimerosal-containing vaccines reported by Pichichero et al. (2002).

An important consequence of the difference in blood half-lives is the remarkable accumulation of blood Hg during repeated exposure to MeHg. Although the initial blood Hg concentration (at 2 days after the first dose) did not differ between the MeHg and thimerosal groups, the peak blood Hg concentration in the MeHg-exposed monkeys rose to a level nearly three times higher than in the thimerosal monkeys after the fourth dose. Furthermore, the blood clearance of total Hg is 5.4-fold higher after im thimerosal than after oral MeHg exposure. The results indicate that for an equivalent level of chronic exposure, the area under the curve of total blood Hg concentrations in human infants receiving repeated im injections of thimerosal-containing vaccines will be significantly lower than that in those exposed chronically to MeHg via the oral route.

A much lower brain concentration of total Hg was observed in the thimerosal monkeys compared with the MeHg monkeys, that is, a 3- to 4-fold difference for an equivalent exposure of Hg. Moreover, total Hg is cleared much more rapidly from the brain after thimerosal than after MeHg exposure (24 vs. 60 days). It appears that the difference in brain Hg exposure between thimerosal and MeHg is largely driven by their differences in systemic disposition kinetics (i.e., the blood level). The average brain-to-blood partitioning ratio of total Hg in the thimerosal group was slightly higher than that in the MeHg group (3.5 ± 0.5 vs. 2.5 ± 0.3, *t*-test, *p* = 0.11). Thus, the brain-to-blood Hg concentration ratio established for MeHg will underestimate the amount of Hg in the brain after exposure to thimerosal.

The large difference in the blood Hg half-life compared with the brain half-life for the thimerosal-exposed monkeys (6.9 days vs. 24 days) indicates that blood Hg may not be a good indicator of risk of adverse effects on the brain, particularly under conditions of rapidly changing blood levels such as those observed after vaccinations. The blood concentrations of the thimerosal-exposed monkeys in the present study are within the range of those reported for human infants after vaccination (Stajich et al. 2000). Data from the present study support the prediction that, although little accumulation of Hg in the blood occurs over time with repeated vaccinations, accumulation of Hg in the brain of infants will occur. Thus, conclusion regarding the safety of thimerosal drawn from blood Hg clearance data in human infants receiving vaccines may not be valid, given the significantly slower half-life of Hg in the brain as observed in the infant macaques.

There was a much higher proportion of inorganic Hg in the brain of thimerosal monkeys than in the brains of MeHg monkeys (up to 71% vs. 10%). Absolute inorganic Hg concentrations in the brains of the thimerosal-exposed monkeys were approximately twice that of the MeHg monkeys. Interestingly, the inorganic fraction in the kidneys of the same cohort of monkeys was also significantly higher after im thimerosal than after oral MeHg exposure (0.71 ± 0.04 vs. 0.40 ± 0.03). This suggests that the dealkylation of ethylmercury is much more extensive than that of MeHg.

Previous reports have indicated that the dealkylation of Hg is a detoxification process that helps to protect the central nervous system (Magos 2003; Magos et al. 1985). These reports are largely based on histology and histochemistry studies of adult rodents exposed to Hg for a short period of time. The results of these studies indicated that damage to the cerebellum was observed only in MeHg-treated animals that had much lower levels of inorganic Hg in the brain than animals comparably treated with ethylmercury. Moreover, the results did not indicate the presence of inorganic Hg deposits in the area where the cerebellar damage was localized (granular layer).

In contrast, previous studies of adult *M. fascicularis* monkeys exposed chronically to MeHg have indicated that demethylation of Hg occurs in the brain over a long period of time after MeHg exposure and that this is not a detoxification process (Charleston et al. 1994, 1995, 1996; Vahter et al. 1994, 1995). Results from these studies indicated higher inorganic Hg concentrations in the brain 6 months after MeHg exposure had ended, whereas organic Hg had cleared from the brain. The estimated half-life of organic Hg in the brain of these adult monkeys was consistent across various brain regions at approximately 37 days (similar to the brain half-life in the present infant monkeys). The estimated half-life of inorganic Hg in the brain in the same adult cohort varied greatly across some regions of the brain, from 227 days to 540 days. In other regions, the concentrations of inorganic Hg remained the same (thalamus) or doubled (pituitary) 6 months after exposure to MeHg had ended (Vahter et al. 1994, 1995). Stereologic and autometallographic studies on the brains of these adult monkeys indicated that the persistence of inorganic Hg in the brain was associated with a significant increase in the number of microglia in the brain, whereas the number of astrocytes declined. Notably, these effects were observed 6 months after exposure to MeHg ended, when inorganic Hg concentrations were at their highest levels, or in animals solely exposed to inorganic Hg (Charleston et al. 1994, 1995, 1996). The effects in the adult macaques were associated with brain inorganic Hg levels approximately five times higher than those observed in the present group of infant macaques. The longer-term effects (> 6 months) of inorganic Hg in the brain have not been examined. In addition, whether similar effects are observed at lower levels in the developing brain is not known. It is important to note that “an active neuroinflammatory process” has been demonstrated in brains of autistic patients, including a marked activation of microglia (Vargas et al. 2005).

The American Academy of Pediatrics and the U.S. Public Health Service (1999) published a joint statement that urged “all government agencies to work rapidly toward reducing children’s exposure to mercury from all sources.” The statement recommended that thimerosal be removed from vaccines as soon as possible as part of this overall process. Between 1999 and 2001, vaccines currently recommended for children ≤ 6 years of age were made available in thimerosal-free formulations in the United States (Centers for Disease Control and Prevention 2001). Exposures to thimerosal through pediatric vaccines, however, still occur in other countries where multiple-dose vials are used to maintain childhood immunization programs and the control of preventable disease (Knezevic et al. 2004).

Recent publications have proposed a direct link between the use of thimerosal-containing vaccines and the significant rise in the number of children being diagnosed with autism, a serious and prevalent developmental disorder (for review, see IOM 2001). Results from an initial IOM review of the safety of vaccines found that there was not sufficient evidence to render an opinion on the relationship between ethylmercury exposure and developmental disorders in children (IOM 2001). The IOM review did, however, note the possibility of such a relationship and recommended further studies be conducted. A recently published second review (IOM 2004) appears to have abandoned the earlier recommendation as well as backed away from the American Academy of Pediatrics goal. This approach is difficult to understand, given our current limited knowledge of the toxicokinetics and developmental neurotoxicity of thimerosal, a compound that has been (and will continue to be) injected in millions of newborns and infants.

The key findings of the present study are the differences in the disposition kinetics and demethylation rates of thimerosal and MeHg. Consequently, MeHg is not a suitable reference for risk assessment from exposure to thimerosal-derived Hg. Knowledge of the biotransformation of thimerosal, the chemical identity of the Hg-containing species in the blood and brain, and the neurotoxic potential of intact thimerosal and its various biotransformation products, including ethylmercury, is urgently needed to afford a meaningful interpretation of the potential developmental effects of immunization with thimerosal-containing vaccines in newborns and infants. This information is critical if we are to respond to public concerns regarding the safety of childhood immunizations.

## Correction

In the original manuscript published online, there were two errors that have been corrected here. First, in the Abstract, the standard errors for the average brain-to-blood concentration ratios were incorrect for thimerosal and MeHg-exposed monkeys. Second, in the last paragraph of “Data analysis,” the statement about the two concentration measures has been corrected to “the two concentration measures (e.g., blood and brain) did not decline in parallel with time.”

## Figures and Tables

**Table 1:** Study design and schedule.

	Age (days)																		
	0 (birth)	2	4	7	9	11	14	16	18	21	23	25	28	31	35	38	42	45	49
MeHg group (oral dose, μg/kg)	20			20			20			20									
				OPV (0)						OPV (0)									
Thimerosal group [ethylmercury dose, in im vaccine (μg/kg)]	OPV (0)			HB (4)			OPV (0)			HB (4)									
	HB (20)			DTP (8)			DTP (10)			DTP (8)									
				Hib (8)			Hib (10)			Hib (8)									
Blood draws (days after most recent dose)	0	2	4	7	2	4	7	2	4	7	2	4	7	10	14	17	21	24	28
Sacrifice day (days after last dose)											2	4	7						28

Abbreviations: DTP, diphtheria/tetanus/pertussis vaccine; HB, hepatitis B vaccine; HIB, haemophilus influenzae type b vaccine; OPV, oral polio vaccine.

**Table 2:** Mean ± SE body and brain weight and brain-to-body weight ratio at sacrifice.

Exposure group	Body weight (g)	Brain weight (g)	Brain:body weight ratio
Controls (*n* = 9)	509.3 ± 52.0	52.1 ± 2.5	0.107 ± 0.009
MeHg exposed (*n* = 17)	499.1 ± 17.5	51.1 ± 1.1	0.103 ± 0.003
Thimerosal exposed (*n* = 17)	529.1 ± 25.4	52.7 ± 1.2	0.102 ± 0.003

**Table 3:** Parameter estimates derived from a one-compartment analysis of the mean blood total Hg concentration for the oral MeHg group (*n* = 17).

Model parameters	Mean ± SD
*V*/*F* (L/kg)	1.67 ± 0.07
*k*_*a*_ (day^−1^)	2.07 ± 1.04
*K* (day^−1^)	0.0276 ± 0.0024
*T*_1/2_ (days)	21.5
Cl/*F* (mL/day/kg)	46.1

**Table 4:** Parameter estimates derived from a two-compartment analysis of the mean blood total Hg concentration for the im thimerosal group (*n* = 17).

Model parameters	Mean ± SD
*k*_*a*_ (day^−1^)	3.24 ± 3.00
*k*_12_ (day^−1^)	0.081 ± 0.076
*k*_21_ (day^−1^)	0.177 ± 0.138
*k*_10_ (day^−1^)	0.148 ± 0.024
*T*_1/2,α_ (day)	2.13
*T*_1/2,β_ (day)	8.62
*V*_*c*_/*F* (L/kg)	1.68 ± 0.30
*V*_*ss*_/*F* (L/kg)	2.45
*V*_*p*_ (L/kg)	0.77
Cl/*F* (mL/day/kg)	248
